# Usefulness of 3D slicer for the planning and monitoring of hepatocellular carcinoma treatment using FUS

**DOI:** 10.1186/2050-5736-3-S1-O84

**Published:** 2015-06-30

**Authors:** Nobutaka Doba, Hiroyuki Fukuda, Kazushi Numata, Ayako Takeda, Yoshiharu Hao, Akito Nozaki, Makoto Chuma, Masaaki Kondo, Shin Maeda, Shigeo Takebayash, Akira Kobayashi, Juichi Tokuda, Katsuaki Tanaka

**Affiliations:** 1 Yokohama City University, Yokohama, Japan

## Background/introduction

FUS is a noninvasive treatment method, as complete coagulation necrosis is achieved without the insertion of any instruments. However, FUS monitor has the poor visualization because of the presence of the multi-reflections, rib shadows and the emergence of the hyperecho after the FUS treatment. 3D Slicer imaging is a diagnostic imaging support system that can provide the same cross-sectional MPR images on the same monitor screen using DICOM volume data from MRI which are not influenced by those artifacts. The purpose of this study was to utilize an interventional navigation system designed for FUS assisted by 3D Slicer was proposed, and a phantom study was carried out to assess the proposed system.

## Methods

The FUS system (Mianyang Haifu Tech) was used under ultrasound guidance. In this system, the open-source navigation software is connected together images using an open network communication protocol, OpenIGTLink. A Polaris Vicra optical tracker (Northern Digital, Ontario, Canada) was used. MRI scans (Signa HDX 3.0T system; GE Healthcare) were performed, and 3D Slicer was customized to combine MR images for the navigation.

Testing was performed using an abdominal phantom (CIRS Model057, Norfolk, VA).

## Results and conclusions

3D slicer could make the multiplanar reconstruction images of MRI displayed in the same sections of US. The synchronous movements of the same sections of US and MRI were shown in real time. Performance tests of phantom show that the registration error of the system was 2.2 ± 1.8 mm within the liver (n=12). Conclusion: 3D Slicer imaging is useful for FUS treatment for HCC, compensating for the occasionally poor visualization provided by US monitor.

**Figure 1:**
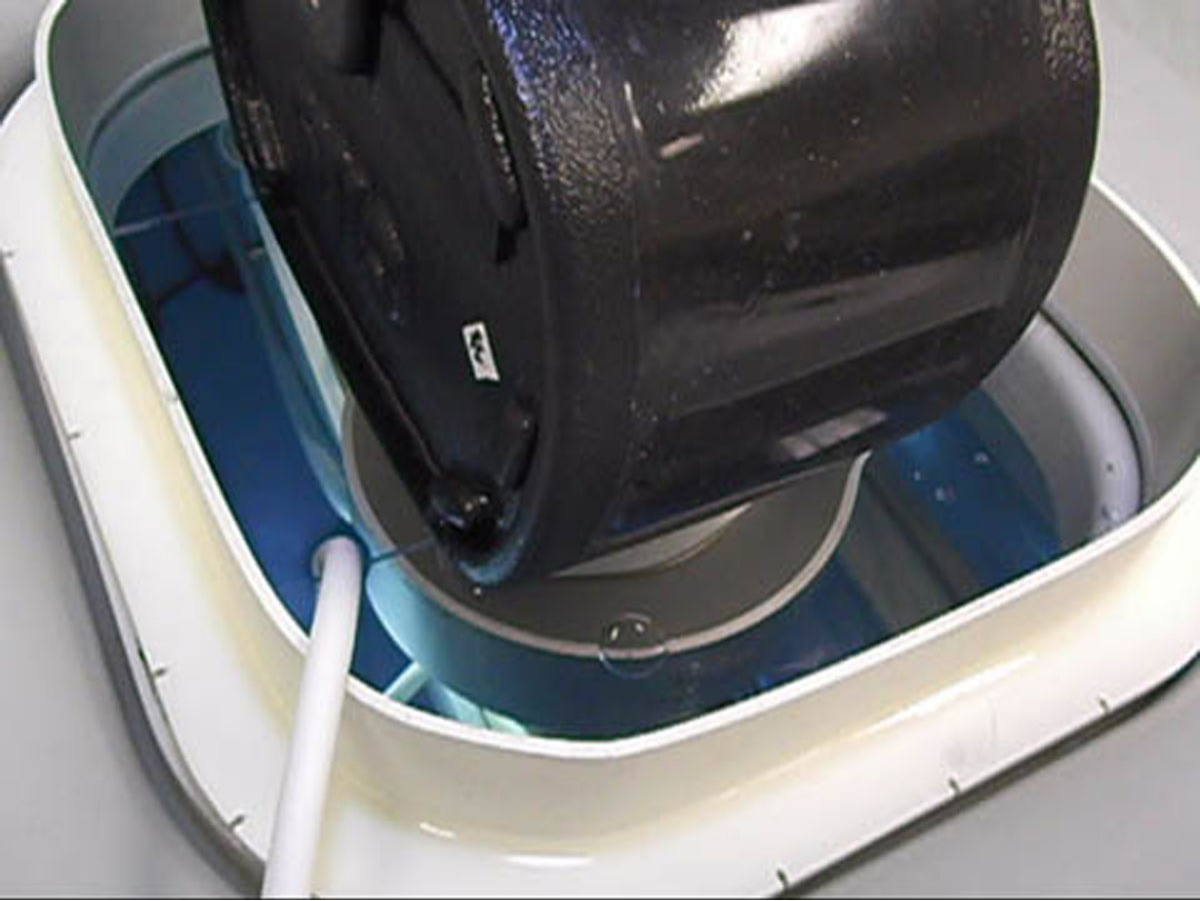
The tumor therapy system. A 2-5 MHz imaging probe was positioned in the center of the FUS transducer and was mounted in a reservoir of degassed water. Phantom was positioned in contact with the degassed water.

**Figure 2:**
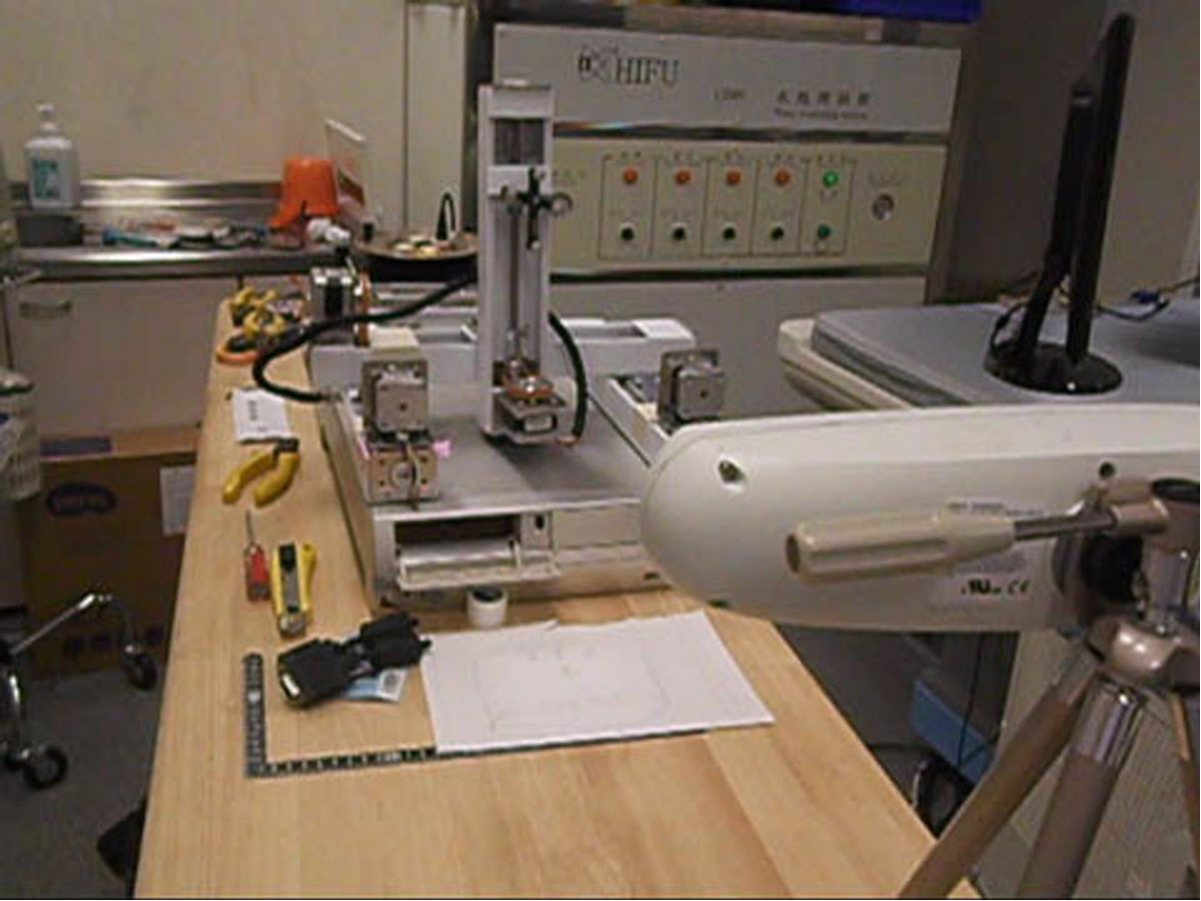
An optical tracker (Northern Digital, Ontario, Canada) was attached outside of a reservoir of degassed water and followed by Polaris Vicra.

**Figure 3:**
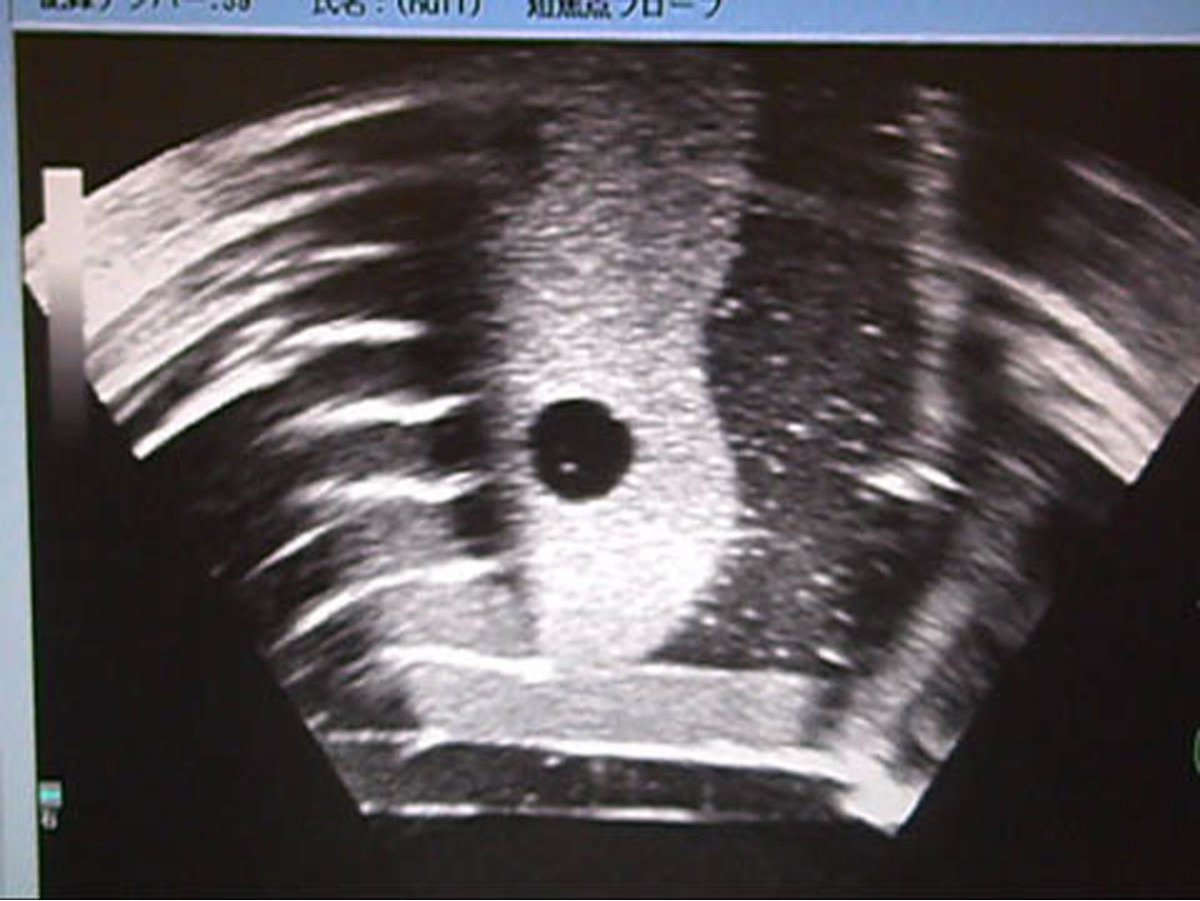
The cystic lesion of the phantom was depicted in the US monitor of the tumor therapy system.

**Figure 4:**
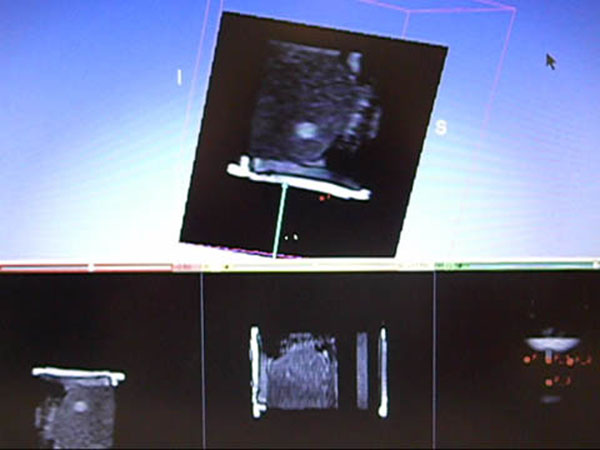
3D slicer image of MRI with high intensity area was displayed in a manner resembling conventional monitor US to assist the FUS treatment.

